# Adaptation of the Turkish version of the obsession with COVID-19 scale (TR-OCS): its association with psychological distress and resilience

**DOI:** 10.1186/s41155-022-00242-y

**Published:** 2022-12-20

**Authors:** Aslı Kartol, Osman Söner

**Affiliations:** 1 Department of Psychological Counseling and Guidance, Faculty of Education, Gaziantep University, Gaziantep, Turkey; 2 Department of Psychological Counseling and Guidance, Faculty of Education, Uludağ University, Bursa, Turkey

**Keywords:** COVID-19, Obsession; adaptation, Turkish version, OCS, Psychological distress

## Abstract

The COVID-19 pandemic adversely affected the physical and mental health of individuals. Measures required to prevent the spread of the virus, such as isolation, hygiene, mask use, and lockdown, led individuals to develop obsessive thoughts and behaviors. This study aims to adapt the obsession with COVID-19 scale (OCS) to Turkish culture. This four-item scale aims at measuring obsessive thoughts related to COVID-19. The study group consisted of 870 participants from seven regions of Turkey. Data from different groups were collected for exploratory and confirmatory factor analyses. Data for the exploratory factor analysis were collected from 296 (71.5%) females and 118 (28.5%) males (*n* = 414). For the confirmatory factor analysis, data were collected from 301 (66.0%) females and 155 (34.0%) males (*n* = 456). As a result of the analysis, the one-dimensional structure of the TR-OCS was confirmed in the Turkish sample. It was revealed that TR-OCS scores had a positive relationship with depression, anxiety, and stress but a negative relationship with resilience. The findings confirmed that the TR-OCS was valid and reliable. This scale is a short and effective tool for measuring obsessive thoughts related to COVID-19.

## Introduction

The COVID-19 pandemic first emerged in Wuhan, China, in December 2019, and the World Health Organization declared it a global pandemic on January 30, 2020. In addition to its effects on physical health, coronavirus has become a massive and global threat to psychological health (Talevi et al., 2020). The COVID-19 pandemic is a significant public health crisis that has had devastating effects on people worldwide (Arslan et al., 2020). This pandemic is an unprecedented public health challenge with severe consequences on social life and the economy (United Nations Development Programme, 2020). In this sense, the coronavirus disease not only led to the risk of death but also triggered significant psychological problems (Ahorsu et al., 2020). This, in turn, resulted in strict measures being taken in the countries where the COVID-19 pandemic spread.

During the COVID-19 pandemic, healthcare and safety professionals, social media networks, etc. specifically emphasized the effects of personal hygiene on the virus. However, the social pressure to be hygienic can cause certain disorders such as hypersensitivity, excessive behaviors, and subsequently obsessive-compulsive disorder (OCD). Moreover, it can intensify the available anxiety and OCD symptoms (Adibi et al., 2020). Similarly, Jelinek et al. (2021) stated that pandemic measures affected people with OCD, especially those with compulsive handwashing. OCD patients expressed that they had valid reasons for their fear of contamination. As there is no explicit evidence as to when the pandemic will end, mental health interventions during and after the pandemic are of vital importance.

Cullen et al. (2020) underlined that measures taken against the pandemic could exacerbate the already existing mental health problems and cause symptoms in those who did not have any mental disorder. Under normal conditions, the fear of COVID-19 is an essential and desired force for the measures to prevent the spread of the disease. However, a very high level of fear can result in a psychiatric disorder (Adibi et al., 2020). The pandemic killed millions of people and had significant psychological impacts on the emotions, thoughts, and behaviors of those who lost their loved ones. They were very prone to suffering from obsessive thoughts about death (Arslan, 2021). Obsessive thoughts, reflected in behaviors, penetrate all aspects of life and disrupt daily routines. Therefore, further research should focus on examining the effects of the COVID-19 pandemic on people and reducing its adverse effects on mental health (Yıldırım & Güler, 2021).

Banerjee (2020) found that obsessive-compulsive behaviors and hoarding behaviors appeared during the pandemic. Vos (2021) pointed out that such behaviors included washing hands repeatedly and wearing a mask and gloves and even a laboratory suit while going outside. During the pandemic, people also began to stock food, protective equipment, and other materials. In this sense, thoughts and behaviors went to extreme limits and led to psychological disorders. The COVID-19 pandemic negatively affected both children and adults who had to confront many challenging feelings (Javed et al., 2020). During the COVID-19 pandemic, a remarkable increase was observed in the number of mental disorders, especially those with depression and anxiety symptoms (López-Núñez et al., 2021; Song et al., 2021; Talevi et al., 2020). Bueno-Notivol et al. (2021) revealed in their meta-analysis study that the depression rate was seven times higher during the pandemic, proving the severe consequences of the COVID-19 pandemic on mental health. Therefore, in addition to various efforts to prevent the spread of the disease and other related problems, psychological crisis interventions are of great importance (Talevi et al., 2020). Obsessive thoughts are unwanted thoughts that emerge suddenly in an out-of-control manner and cause stress. No matter how hard one tries to control their obsessive thoughts, they may fail. Even if there is some relief from time to time, this is usually momentary and lasts only a short time (Purdon & Clark, 2005). Since the COVID-19 process is an unexpected, long process causing heavy losses, even if the pandemic ends, certain physical symptoms are likely to raise suspicion about whether one is infected or not. This can trigger the person’s fear and obsessive thoughts. Concerning this subject, Skalski et al. (2020) revealed that constantly thinking about COVID-19 was associated with increased trauma and fear of COVID-19.

Taking these as a starting point, the study aimed to adapt to Turkish the “Obsession with COVID-19 Scale,” which was developed by Lee (2020). By doing so, the study sought to determine the harmful effects of the COVID-19 pandemic on the mental health of the Turkish sample. This scale has already been adapted to several cultures such as Korean (Choi et al., 2020), Portuguese (Andrade et al., 2021), Persian (Asanjarani et al., 2021), Urdu (Ashraf et al., 2020), and Spanish (Caycho-Rodríguez et al., 2021). In this study, we tested whether the one-dimensional factor structure of the OCS scale was suitable for the Turkish sample. We assumed that this scale had a one-dimensional structure in Turkish culture, too, and could have an acceptable internal consistency. It was also assumed that women might be more obsessed with COVID-19 than men. Also, individuals with high levels of depression, anxiety, and stress were assumed to have an increased obsession with COVID-19, while those with higher levels of psychological resilience may be less obsessed with COVID-19. The *COVID-19 fear scale* (Ladikli et al., 2020; Satici et al., 2021) and the *COVID-19 anxiety scale* (Karaahmet et al., 2021) were adapted to Turkish. The present study is the first in the relevant literature in that it not only conducts the validation of the scale on a Turkish sample but also, at the same time, examines the relationships among depression, anxiety, stress, and resilience.

## Methods

### Participants

Data were collected from a total of 870 participants. Data from different groups were collected for exploratory and confirmatory factor analyses. Firstly, data for the exploratory factor analysis were collected from 296 (71.5%) females and 118 (28.5%) males. In the second stage, confirmatory factor analysis was performed with the data collected from 301 (66.0%) females and 155 (34.0%) males. The average ages of the participants in the first and second analyses were 31.31 ± 9.50 and 31.08 ±10.56, respectively. Table 1 below presents certain demographic characteristics of the two groups

**Table 1:** Demographic characteristics of the participants

Demographic characteristics	Group 1		Group 2		Total sample	
	***n***	%	***n***	%	***n***	%
Gender						
Female	296	71.5	301	66.0	597	68.6
Male	118	44.709	155	34.0	273	31.4
Educational Background						
Primary school	125	30.2	145	44.804	270	31.0
Middle School	49	44.784	76	44.758	125	44.665
High school	76	44.669	83	44.610	159	44.638
Undergraduate	85	44.701	78	44.578	163	44.760
Postgraduate	79	44.580	74	44.608	153	44.729

### Measures

The study instruments included “The Obsession with COVID-19 Scale (OCS),” “The Depression, Anxiety, and Stress Scale-Short Form (DASS-21),” and “The Brief Resilience Scale (BRS).” Besides, the researchers developed a personal information form to collect data related to certain demographic characteristics (e.g., gender, age, and educational background).

### *The obsession with* COVID-19 scale (OCS)

It was developed by Lee (2020) to determine the level of obsessive thinking about COVID-19. The scale consists of four 5-point Likert-type items (0 = not at all, 1 = rare, less than a day or two, 2 = several days, 3 = more than 7 days, 4 = nearly every day over the last 2 weeks). In the original scale development process, Lee (2020) demonstrated its reliability and validity on two large samples (*N* = 775; *N* = 398) of adults. The reliability was 0.84 and 0.85, respectively.

### The depression, anxiety, and stress scale-*s*hort *f*orm (DASS-21)

The DASS-21 was developed by Lovibond and Lovibond (1995) to determine depression, anxiety, and stress levels. There were 42 items in the original version, but the number of items was later reduced to 21. Yılmaz et al. (2017) adapted the 21-item short form into Turkish. It is a 4-point Likert-type scale, where the lowest and highest scores for each dimension are 7 and 35, respectively. The scale items are scored as 0—never, 1—sometimes, 2—quite often, and 3—always. In the adaptation process, Yılmaz et al. (2017) found that the scale still had a three-factor structure in the Turkish sample, and the goodness-of-fit values were within acceptable ranges *(χ*^2^/*df* = 2.84, *GFI* = 0.99, *AGFI* = 0.98, *RMR* = 0.05, *NFI* = 0.98, *RMSEA* = 0.05, *SRMR* = 0.05). Cronbach’s alpha internal consistency coefficient was 0.82 for the depression subdimension, 0.81 for the anxiety subdimension, and 0.75 for the stress subdimension. In the current study, Cronbach’s alpha reliability coefficient was 0.85 for the depression subdimension, 0.84 for the anxiety subdimension, and 0.84 for the stress subdimension.

### Brief *r*esilience *s*cale (BRS)

The 5-point Likert-type scale was developed by Smith et al. (2008) to determine agents’ psychological resilience levels. There are six items on the scale. It was adapted to Turkish by Doğan (2015). In the adaptation process, Doğan (2015) found that the scale still had a single-factor structure in the Turkish sample, and the goodness-of-fit values were within acceptable ranges (*χ*^2^/*df* = 1.83, *NFI* = 0.99, *NNFI* = 0.99, *CFI* = 0.99, *IFI* = 0.99, *RFI* = 0.97, *GFI* = 0.99, *AGFI* = 0.96, *RMSEA* = .05, *SRMR* = 0.03). Cronbach’s alpha internal consistency coefficient was 0.83. In the current study, Cronbach’s alpha reliability coefficient was 0.88.

### Procedure

From adults living in different regions of Turkey, data collection was carried out online using Google Forms. The link to the survey was posted on Turkish online forums and social networking communities (e.g., Facebook, WhatsApp) between 12 March 2021 and 12 June 2021. Using convenience sampling, participants were recruited in the research on a voluntary basis. No limitation was imposed on any variable other than the age limit of being 18 years or older. In current study, the back translation method was used. The OCS was first translated into Turkish by three field professors and then translated back into English by three linguists. Finally, the researchers finalized the Turkish version of OCS (TR-OCS). Study procedures were designed considering the Declaration of Helsinki. Informed consent forms were obtained from the participants. Since the data was collected online and responding to all the items was required, there was no missing data. Necessary permission was obtained from the author of the original scale. Ethical approval was granted by Gaziantep University Ethics Committee (ethics number: 163279).

### Data analysis

Exploratory factor analysis was performed to determine the item factors and factor loadings in the OCS. The principal component analysis method was used in this study to reveal the factor structure. Considering its advantages in exploratory factor analysis compared to other methods, principal component analysis is predominantly preferred to reveal factor structures (Stevens, 2009). Later, confirmatory factor analysis was applied to determine the model fit of the structure described in exploratory factor analysis (Kahn, 2006). In this study, the criteria that we used to evaluate the good fit of the model are determined as CMIN/DF (0 ≤ *χ*^2^/*df* ≤ 2), GFI (0.95 ≤ *GFI* ≤ 1.00), CFI (0.97 ≤ *CFI* ≤ 1.00), AGFI (0.90 ≤ *AGFI* ≤ 1.00), RMSEA (0 ≤ *RMSEA* ≤ .05), NFI (0.95 ≤ *NFI* ≤ 1.00), TLI (0.97 ≤ *TLI* ≤ 1.00), and SRMR (0 ≤ *SRMR* < .05) (Schermelleh-Engel et al., 2003). Maximum likelihood (ML) was used as the estimation method in confirmatory factor analysis. Exploratory and confirmatory factor analyses were carried out with data collected from two groups. Firstly, in order to determine the factor structure and external validity of the scale, the data of 442 participants were used. The data of 28 participants were excluded from the analysis as they were extreme values in terms of the Mahalanobis distance method. Later, explanatory factor analysis was performed on the remaining 414 data using the SPSS program. In the second stage, data were collected from 476 participants for confirmatory factor analysis. However, the data of 10 participants were excluded from the analysis with extreme values according to the Mahalanobis distance method. In both analyses, kurtosis and skewness values had a normal distribution between −1.5 and +1.5 (Tabachnick & Fidell, 2013). Cronbach’s alpha coefficients were measured to determine the internal consistency and reliability of the scales. In addition, Pearson correlation analysis was performed to determine the relationships between the ages of the participants and their scores. Correlation values between .00 and 0.30 are interpreted as low, between 0.30 and 0.70 as medium, and above 0.70 as high correlation (Büyüköztürk et al., 2016).

## Results

### Exploratory factor analysis

Exploratory factor analysis was performed to ensure the construct validity of the OCS. The Barlett test and Kaiser-Meyer-Olkin (KMO) values were examined to determine whether the data set was suitable for factor analysis. The Barlett test results indicated a significant value [*χ*^2^ *(df* = 6) = 375.991, *p* < .001], and the KMO value was at a reasonable level of 0.707. It was seen that the data set was suitable for factor analysis. Then, eigenvalue statistics were checked to determine the number of factors. Table 2 demonstrates the findings regarding the eigenvalue statistics, item means, kurtosis, skewness, and factor load values.

**Table 2:** Descriptive statistics and factor loading values of the scale items (*n* = 414)

Items	Mean	SD	Skewness	Kurtosis	Item-total correlation	Factor load
1. I had disturbing thoughts that I may have caught the coronavirus	1.15	1.21	0.823	−0.21	0.54	0.65
2. I had disturbing thoughts that certain people I saw may have the coronavirus	1.69	1.30	0.25	−1.00	0.56	0.80
3. I could not stop thinking about the coronavirus	1.39	1.25	0.59	−0.67	0.49	0.67
4. I dreamed about the coronavirus	1.35	0.77	0.56	0.77	0.31	0.43
Eigenvalues						1.700
Explained variance (%)						42.50
Explained total variance						42.50

The relationship of the items with the factors is explained by the factor load value. Although there is no definite limit on the minimum value that an item must reach in order to enter any factor, 0.30 or 0.40 is generally recommended. No item was removed from the scale because factor loads had factor loads between 0.43 and 0.80. In general, a load value between 0.30 and 0.59 is considered medium, and those above 0.60 are considered high. In this study, only one item had a medium factor load of 0.43, while the others had a high factor load of 0.65–0.80. These values were found to be sufficient as an indicator of the construct validity of the scale. As a result, no item was excluded or no changes were made as it still had a single-factor structure. Thus, the 42.50% obtained in this study indicate a high percentage of variance explained. Also, kurtosis and skewness values of the scale items generally show a normal distribution between −1 and +1, and the arithmetic means and standard deviations are close to each other.

### Confirmatory factor analysis

Confirmatory factor analysis was performed to test the single-factor structure of the OCS. Table 3 below presents the confirmatory factor analysis results regarding the goodness-of-fit index.

**Table 3:** Model fit indices

	***χ***^**2**^/df	GFI	CFI	AGFI	RMSEA	NFI	TLI	SRMR
Before covariance	3.456	0.751	0.746	0.675	.089	0.678	0.754	.04
After covariance	.058	1.00	1.00	0.999	.000	1.00	1.00	.002

**Fig. 1:**
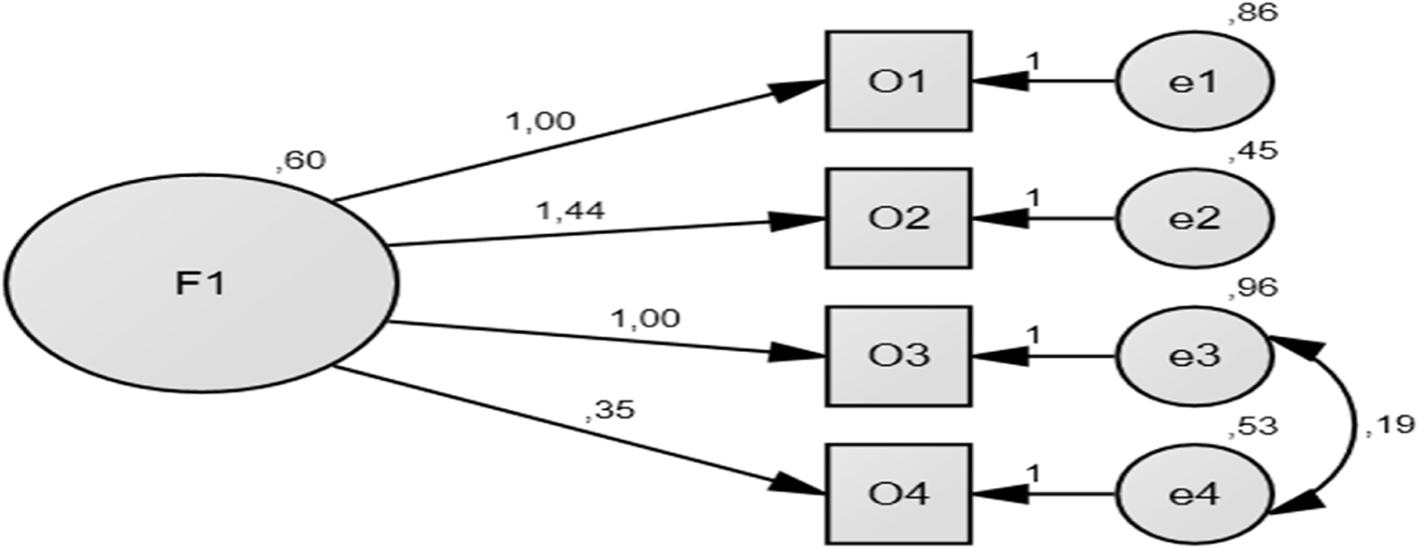
Unstandardized factor loadings for the final model

When the data in Table 3 are examined, it is seen that goodness-of-fit values are not in the acceptable range before modification. After adding the error covariance, goodness-of-fit values were found in the acceptable range. At this point, *χ*^2^/df value was compatible with the data. In addition, *GFI* = 1.00, *AGFI* = 0.999, and *SRMR* = .002 values, which are the fit indices of the residues, are in acceptable level; *NFI* = 1.00, *CFI* = 1.00, and *TLI* = 1.00, which are the fit indices of the independent model, have a good fit; the RMSEA value, which is the mean square root of the approximate errors, was in acceptable level. In light of the findings, the single-factor structure of the scale was confirmed. Figure 1 below shows the final model of fit indices for confirmatory factor analysis.

As shown in Fig. 1, unstandardized factor loadings ranged between 0.35 and 1.44, which were all significant at the .01 level. Besides, standard error values ranged between 0.45 and 0.96. Modification indices (MI) values give an idea about the change in the chi-square value corresponding to one degree of freedom (df). High MI values are interpreted as a link between the variables. To be estimated freely, each variable should have MI values of “0.” However, this is the ideal situation, especially in the social sciences, where MI values are unlikely to be zero. The covariance between two variables with high MI values will help reduce the chi-square value, thus making the model more fit. Therefore, in this model, a covariance was created between e3 and e4 error values to fit the MI values.

### Reliability

The relationship between the items of the adapted scale was examined with the Cronbach’s alpha reliability coefficient. The findings were obtained from these reliability estimates in the Table 4. Cronbach’s alpha for four items was found 0.733. Table 4 presents the inter-item correlation matrix and the alpha if each item was deleted.

**Table 4:** Inter-item correlation matrix

	Cronbach’s alpha if item deleted	1	2	3	4
1. I had disturbing thoughts that I may have caught the coronavirus	0.673	1			
2. I had disturbing thoughts that certain people I saw may have the coronavirus	0.603	0.551	1		
3. I could not stop thinking about the coronavirus	0.637	0.396	0.531	1	
4. I dreamed about the coronavirus	0.745	0.232	0.299	0.415	1

According to the findings in the Table 4, the correlation between the fourth item and the first and second items is below 0.30. If the fourth item is removed from the analysis, the reliability coefficient of the adapted scale will be 0.745, which is not much different from the initial value. For these reasons, the item was not excluded from the scale.

### Evidence of validity based on relations to other variables

The DASS-21 and the brief resilience scale, whose construct validity had been tested on the Turkish sample, were used to meet the external validity criterion of the OCS. Besides, Pearson correlation analysis was performed to determine the relationships between the participants’ ages and their scores. Table 5 presents the results of descriptive statistics and correlation analysis.

**Table 5:** Descriptive statistics and correlation analysis results

	***N***	***M***	df	Skew	Kurt	1	2	3	4	5	6
1. Anxiety total	414	46.174	44.777	0.65	−.07	1					
2. Depression total	414	44.599	33.695	0.44	−0.40	0.74*	1				
3. Stress total	414	29.068	33.695	0.17	−0.48	0.73*	0.79*	1			
4. Obsession with COVID-19 total	414	21.276	16.132	0.62	−0.15	0.34*	0.29*	0.34*	1		
5. Resilience total	414	19.49	24.959	−0.28	−0.12	−0.43*	−0.50*	−0.53*	−0.23*	1	
6. Age	414	44.651	19.603	.89	0.66	−0.11*	−0.13*	−0.13*	−.05	0.16*	1

*Correlation is significant at the 0.05 level (2-tailed)

As seen in Table 5, there was a positive moderate correlation between the OCS and anxiety and stress subdimensions of DASS-21. There was also a slightly positive correlation between the OCS and depression. Moreover, there was a negative relationship between resilience and the OCS. The findings showed that the OCS, whose construct validity was previously tested on the Turkish sample, met the external validity criteria.

Additionally, age was found to have a negative correlation with anxiety, depression, and stress but positive relationship with resilience. However, no significant relationship was found between age and the OCS.

## Discussion

The main purpose of this research was to test the factor structure, validity, and reliability of the OCS and to adapt it to Turkish. Based on the analysis results, the one-dimensional structure of the TR-OCS was confirmed in Turkish culture. Furthermore, the TR-OCS had acceptable levels of internal consistency and reliability. Analyses for the external criterion validity showed that OCS positively correlated with depression, anxiety, and stress but negatively correlated with psychological resilience. Hence, these findings proved the external criterion validity of the TR-OCS. This adaptation study produced findings that are expected to further contribute to the universality of the OCS (Andrade et al., 2021; Asanjarani et al., 2021; Ashraf et al., 2020; Choi et al., 2020), which has already been adapted to different countries.

Obsession with COVID-19 positively correlated with depression, anxiety, and stress. On the other hand, there were negative correlations between obsession and resilience. These results are in line with the assumptions of the research. Many studies in the literature have investigated the depression, anxiety, and stress levels of individuals during the COVID-19 pandemic (Arafa et al., 2021; Feng et al., 2020; Gallagher et al., 2020; Lakhan et al., 2020; Satici et al., 2021; Stanton et al., 2020; Verma & Mishra, 2020; Yıldırım & Güler, 2021; Zandifar et al., 2020). In the study adapting the OCS scale to Urdu, OCS scores were not found to be correlated with depression or anxiety. Hence, the authors noted this as a limitation of the study, stating that the study had limited construct validity (Ashraf et al., 2020). There are also studies on psychological resilience during the COVID-19 pandemic (Bozdağ & Ergün, 2021; Flaherty & Nasir, 2020; Killgore et al., 2020; Ran et al., 2020). It is vital to increase the number of studies investigating the long-term psychological effects of the pandemic on people (Talevi et al., 2020). Skalski et al. (2020) revealed that thinking about COVID-19 was not functional, was related to the effects of anxiety and trauma, and negatively affected mental health. Considering the results of these studies, psychological resilience plays a protective role in the COVID-19 process. Therefore, the physiological effects of the virus on the recovery process might be overcome by using correct coping strategies. Increasing depression, anxiety, and stress levels during the COVID-19 process negatively affected the mental health of people, regardless of whether they were infected or not. In this context, dysfunctional thoughts about the COVID-19 process also trigger depression and anxiety in individuals.

Considering the physiological effects of the COVID-19 process, the symptoms differ from person to person as the virus mutates. In this sense, many people contracted the disease even though they were already vaccinated against the virus. Easing the measures can still be a cause for concern for some people. Obsessed individuals may find it compelling to accept the idea that this virus, which has been in our lives for a long time, completely disappeared. In this respect, the data from this scale is important to prevent these dysfunctional thoughts from affecting the mental health of the person and to determine whether different psychological disorders are caused by these thoughts. Although it remains unclear whether the coronavirus pandemic will start over again, preventive studies can be a source of strength for individuals to cope with the virus in the future.

### Limitations

Research results are limited to the data that were collected online from a nonclinical sample. Besides, they are limited to self-report tools. Since the data collection period was 3 months, changes in infection and death rates may have affected the mental health of the general population. Another limitation of the study is that it was not determined whether the sample group was infected with COVID-19 or not. This may make a difference in individuals’ levels of obsession with COVID-19. The study was carried out only on individuals over the age of 18. The COVID-19 pandemic is still an ongoing process. Our research findings are expected to contribute to future studies related to COVID-19 and different psychological factors. The data obtained using the COVID-19 obsession scale can be used in future psychological interventions. Even if the COVID-19 pandemic is over, obsessive thoughts about the pandemic may persist for a while. Therefore, future preventive interventions can examine the relationships between COVID-19 obsession and different psychological variables.

## Conclusion

The TR-OCS is a short and easy scale. The psychometric properties of the TR-OCS are satisfactory. Validity evidence was provided by associations between psychological distress and resilience. Although the pandemic seems to have ended in some countries (measures such as mask use and lockdown have either been eased or lifted), it persists in some countries. The pandemic may re-emerge in the future. In this respect, this scale can contribute to future studies focusing on the relationship between the pandemic and mental health. The scale makes a very important contribution to the literature in detecting dysfunctional thoughts and other factors related to COVID-19. The current study both produced a new mental health scale and offered important data about psychological distress and resilience.

## Data Availability

The original form and data of the COVID-19 obsession scale adapted in the current study are available from the corresponding author upon reasonable request. Also, we have permission from the original creator of the existing instrument or methodology to use and adapt his work in this way.

## Abbreviations

OCS: Obsession with COVID-19 scale
TR-OCS: Turkish version of obsession with COVID-19 scale
OCD: Obsessive-compulsive disorder
DASS-21: Depression anxiety stress scale-21 item
BRS: Brief resilience scale
ML: Maximum likelihood
KMO: Kaiser-Meyer-Olkin
MI: Modification indices
DF: Degree of freedom
